# Comparative Study of the Physicochemical and Microbiological Quality of Liquid, Freeze-Dried, Hot Air-Dried, and Pasteurized Quail Eggs Produced in Benin

**DOI:** 10.1155/2022/1991659

**Published:** 2022-08-10

**Authors:** Kotchikpa Justin Ekpo, Germain Elolo Osseyi, Joseph Dossou, Virgile Ahyi

**Affiliations:** ^1^Regional Center of Excellence in Poultry Sciences (CERSA), University of Lome (UL), BP 1515 Lome, Togo; ^2^College of Biological and Food Techniques (ESTBA), University of Lome, BP 1515 Lome, Togo; ^3^Laboratory of Bioengineering of Food Processes (LABIOPA), Faculty of Agronomic Sciences (FSA), University of Abomey-Calavi, BP 2526 Cotonou, Benin; ^4^Laboratory of Production Processes, Regional Institute of Industrial Engineering and Biotechnology, University IRGIB-AFRICA, 07 BP 231 Cotonou, Benin

## Abstract

Nutrients in quail eggs can be affected by egg product processing technologies. However, freeze-drying would be the most suitable, but it is very costly and difficult to transfer to the quail egg production actors. This study is aimed at comparing the physicochemical and microbiological qualities of liquid, pasteurized, hot air-dried, and freeze-dried quail eggs. Liquid quail eggs were used as controls. The physicochemical and microbiological qualities were evaluated by conventional methods. The results showed that freeze-dried and hot air-dried quail eggs differed (*p* < 0.05) from liquid and pasteurized quail eggs for all the evaluated parameters. On the other hand, pasteurized quail eggs were more concentrated (*p* < 0.05) in dry matter (45.2 ± 0.06%) and fat (25.8 ± 1.33%) with a more basic pH (7.9 ± 0.20) than liquid eggs. As for microbiological parameters, only total mesophilic aerobic germs were present with a significant difference (*p* < 0.05) between liquid (2.0 ± 1.4 log_10_ CFU/g), hot air-dried (2.1 ± 1.5 log_10_ CFU/g), and freeze-dried (1.8 ± 1.0 log_10_ CFU/g) quail eggs, but the load was much lower than the standard (5.7 log_10_ CFU/g). Total coliforms (0 CFU/g), *Enterobacteriaceae* (0 CFU/g), yeasts (0 CFU/g), molds (0 CFU/g), *Salmonella*, and *Staphylococcus aureus* were absent in all the egg products, while the standards provided 2.0 log_10_ CFU/g for total coliforms, 1.0 log_10_ CFU/g for *Enterobacteriaceae*, *Salmonella* absent in 25 g, *Staphylococcus aureus* absent in 1 g, and 2.0 log_10_ CFU/g for yeasts and molds. In conclusion, hot air-drying and pasteurization are transferable and suitable for the processing of quail eggs.

## 1. Introduction

Quail (*Coturnix japonica*) farming is carried out for the production of eggs and meat. The world is increasingly interested in eating quail eggs because of their therapeutic properties and nutritional quality. Japanese quail is one of the most promising sources of sustenance to meet the demand for high-protein eggs [1]. Quail eggs are also known to be rich in essential fatty acids and essential amino acids. However, in Benin, the quail farming sector is faced with multiple problems, the most crucial of which is related to egg conservation. It is important to note that this sector is increasingly being abandoned due to the lack of appropriate conservation technologies for quail eggs. Producers of quail eggs in Benin lose more than 1,688,740 eggs/year, a loss rate of 13.06% [2]. Eggs kept for more than two weeks in an open space lose their quality through breakage or cracks and the infestation of microorganisms within them [3]. According to the *Institut Français d'Eveil Sanitaire* (InAS), the pathogenic microorganisms responsible for Collective Foodborne Toxi-Infections (CFTI), for which eggs and egg products are compromised, are *Salmonella* (85.9%), *Staphylococcus aureus* (7%), *Clostridium perfringens* (2.3%), and other agents (4.4%) [4]. Approximately 90% of human salmonellosis due to the consumption of contaminated eggs or egg products is related to *S. enteritidis* [5]. To limit the risks of damage and contamination related to the fragility of their shells, several studies have been conducted to make concrete proposals to boost the practice of conservation of quail eggs. Hasdar et al. [3] revealed that quail eggs can be preserved by boiling them in water containing salt at a concentration of 30%. This technique does not guarantee a long shelf life of reusable quail eggs as an ingredient. Therefore, to effectively address the preservation problems of quail eggs, Kudre et al. [1] obtained freeze-dried Japanese quail and white leghorn hen egg powders and compared their physicochemical and functional properties. From their results, it was found that freeze-dried quail egg powders were richer in essential amino acids, essential fatty acids, and minerals and less rich in lipids than freeze-dried hen egg powders. Therefore, quail eggs may substitute for chicken eggs by consumer preference. Although freeze-drying is a high-quality preservation technology, it is still costly. Alternative technologies such as hot air-drying and pasteurization could mitigate the cost and promote the transfer of processing technologies to the grassroots. Similarly, freeze-drying, hot air-drying, and pasteurization could also influence the physicochemical and microbiological qualities of liquid quail eggs. To this end, this study sought to compare the physicochemical and microbiological qualities of pasteurized, freeze-dried, and hot air-dried quail eggs using liquid quail eggs as a reference.

## 2. Materials and Methods

### 2.1. Production of Liquid, Pasteurized, Hot Air-Dried, and Freeze-Dried Quail Eggs

The quail eggs in the shell were collected from a quail farmer selected for the best implementation of biosecurity measures. The number of quail eggs required for processing was collected and processed in batches as needed.

The processing of the quail eggs was done in two steps to obtain each egg product:

#### 2.1.1. 1^st^ Step: Preparation of Liquid Quail Eggs

**Figure 1:**
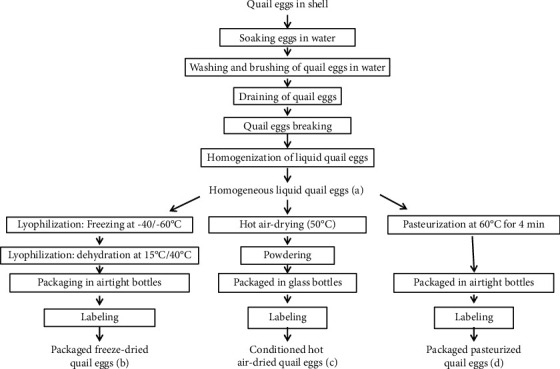
Production diagram of liquid (a), freeze-dried (b), hot air-dried (c), and pasteurized (d) quail eggs.

Common to all technologies, this preparation consisted of weighing the quail eggs in their shells before soaking them in a disinfectant made up of water with a 10% sodium hypochlorite solution for 2 min. The quail eggs that floated and were designated as not fresh were removed from the batch. Fresh eggs were weighed, washed, and brushed. They were then rinsed three times and broken after complete draining in a hygienic and aseptic condition to avoid cross-contamination. The resulting liquid quail eggs were weighed and homogenized with a hand whisk to obtain a uniform liquid. The homogeneous liquid quail eggs were then weighed (Figure 1(a)). A sample of homogeneous quail eggs was collected in sterile, light-tight water pressure bottles for analysis related to liquid quail egg quality. These samples were immediately stored at a temperature of -23°C.

#### 2.1.2. 2^nd^ Step: The Processing Operations

After obtaining the homogeneous liquid quail eggs, the processing operations followed under the following conditions specified for each treatment.

*(1) Production of Freeze-Dried Quail Eggs*. Liquid quail eggs were freeze-dried by the modified method of Truffier and Pinon [6]. Using a freeze-dryer (Group SMD (Society of Management and Diffusion), Division, SERAIL CIRP, Department, Freeze-dryer RP2V), the freezing was carried out at a temperature of -40°C with a maximum of -60°C and dehydration at a pressure of 0.121 mbar for 24 h with a sample of 8 mm thickness or 245 g. The heating temperature was maintained at 15°C with a maximum of 40°C and the lyophilisate at -4.9°C (Figure 1(b)).

*(2) Production of Hot Air-Dried Quail Eggs*. Homogeneous liquid quail eggs were hot air-dried by the method of Ekpo et al. [7]. Hot air-drying was performed at a feed temperature of 50°C for 24 h on a 1 kg liquid quail egg sample by using a halogen cyclone oven (Quigg halogen cyclone oven, model: CO2F, type: AOT-F902) (Figure 1(c)).

*(3) Production of Pasteurized Quail Eggs*. Pasteurization was carried out according to the modified method of USDA [8] in a water bath at a temperature of 60°C for 4 min. The operation was carried out at atmospheric pressure with 383 g of liquid quail eggs. The operation lasted for 1 h and 20 min. To obtain the pasteurized quail eggs, 387 g of water was weighed in the container. The set was placed on a hot plate (Woippy Moselle No. 396033, Ref. No. 4798) that was previously started. The thermometer (probe thermometer, Renkforce) was then introduced into the water to control the water temperature. The water temperature gradually rose to 70°C where 383 g of liquid quail eggs previously weighed in the second container (assuming) was placed on top of the fire. The thermometer was also introduced into the liquid quail eggs to control their temperature. The liquid quail eggs were continuously stirred with a spatula. Upon the introduction of the superimposer, the temperature of the water dropped from 70°C to 48°C and then rose to the temperature of the liquid quail eggs. The latter rose to 60°C and was maintained for 4 min while controlling the water temperature. After 4 min, the superimposer was removed from the water, and the pasteurized quail eggs were weighed and packed in airtight bottles protected from light and humidity (Figure 1(d)).

#### 2.1.3. Packaging and Labeling of Quail Egg Products

The obtained products were packaged in moisture and light-proof glass bottles, while the pasteurized quail eggs were packaged in bottles or glass sealed with moisture and light.

The liquid, pasteurized, hot air-dried, and freeze-dried quail eggs were kept at a temperature of -23°C for three (3) days before proceeding with the different analyses.

### 2.2. Physicochemical and Microbiological Analyses

#### 2.2.1. Sampling

All the samples of each treatment or technology used (liquid, pasteurized, freeze-dried, and hot air-dried packed quail eggs) were mixed, and three samples were randomly selected. These three samples represented the three replicates of each treatment.

#### 2.2.2. Determination of Water Activity

The water activity (a_w_) was determined using an a_w_-meter (Novasina AG, Neuheimstrasse 12, 8853, Lachen, Switzerland). The water activity was measured at a constant temperature of 25°C by placing 10 g of each sample in the apparatus. The water activity value was automatically displayed on the a_w_-meter screen.

#### 2.2.3. Determination of pH

The pH was determined using a pH meter (CRISON pH-meter Basic, 20°) by dissolving 10 g of sample in 50 mL of distilled water [8]. The pH value was automatically displayed on the pH-meter screen.

#### 2.2.4. Determination of Dry Matter

The thermogravimetric method was used with an oven (Memmert) at a temperature of 72°C for 72 h [9].

#### 2.2.5. Determination of Fat Content

The Soxhlet method was used to extract fat with hexane (95%) for 1 hour using the FOSS unit (ST 243, Soxtec) in cellulose cartridges (25 × 60 mm, thickness 2 mm) [10].

#### 2.2.6. Determination of Total Protein Content

Total protein content was determined by the Kjeldahl AOAC 928.08 method [11]. Two grams (2 g) of each quail egg product was weighed and mineralized using a metal heating block (Gerhardt KT20S) with the addition of 15 mL of concentrated sulfuric acid (98%) and two catalysts (Kjeldahl Pastille). Predigestion at 250°C and digestion at 420°C were each carried out for 1 h. The resulting sample was then automatically distilled with 80 mL of 40% concentrated soda and 80 mL of distilled water for 4 min 30 s using a Gerhardt VAP300 distiller. The ammonium was trapped in boric acid (4%). Finally, the distillate was titrated using a SCHOTT TL5000 titrator with the addition of 0.1 N hydrochloric acid. The turning of the indicator indicated the end of the titration. The protein content was determined automatically by multiplying the total nitrogen content by the conversion factor of 6.25.

#### 2.2.7. Determination of Ash Content

This determination consisted of weighing 5 g of each egg product into a crucible. The whole was then placed in a muffle furnace at a temperature of 550°C for 12 h [12]. At the end of this time, the samples were taken out into a desiccator to be weighed. The ash content was finally calculated according to the following formula:

(1) $$\begin{array}{c} \%\text{Cendre}=\frac{\left(\text{MF}-\text{MV}\right)\times 100}{\text{ME}}, \end{array}$$

where MF is the final mass after calcination, MV is the empty mass of the crucible, and ME is the mass of the sample.

### 2.3. Determination of the Microbiological Quality

Microbiological analyses were performed on total aerobic mesophils (TAM), total coliforms, *Enterobacteriaceae*, *Salmonella*, *Staphylococcus aureus*, yeasts, and molds. A suspension was prepared by weighing 10 g of each of the samples (liquid, pasteurized, hot air-dried, and freeze-dried quail eggs) in 90 mL of Tryptone Salt under aseptic conditions. A decimal serial dilution from 10^−1^ to 10^−5^ of this suspension was performed. Then, 1 mL of each dilution was taken for plating the germs in suitable media and conditions. Thus, the total aerobic mesophils were plated on plate count agar at 30°C for 48 h. As for total coliforms and *Enterobacteriaceae*, they were cultured on Violet Red Bile Lactose and Violet Red Bile Glucose, at 44°C and 37°C for 48 h, respectively, while *Staphylococcus aureus* was plated on Baird Parker at 37°C (48 h) after the addition of tellurite (1%) and egg yolk (5%). Mold and yeast counts were performed on Sabouraud supplemented with Chloramphenicol at 30°C after 72 h. For *Salmonella*, 25 g of each sample was preenriched in 225 g of Buffered Peptone Water at 37°C for 24 h. Inoculation was performed by removing 0.5 mL of the preenriched suspension in 9.5 mL of Rappaport Vassiliadis and incubating at 37°C for 24 h. Characteristic colonies were isolated successively on Hektoen and *Salmonella Shigella* agar at 37°C (24 h). After isolation, characteristic colonies were identified on Kligler agar at 37°C for 24 h. Characteristic colonies were confirmed in 0.5 mL of urea at 37°C for 24 h. Two drops of indole were then added to the urea negative colonies. After 5 min, the indole-negative colonies were grown on nutrient agar for 24 h at 37°C to obtain the young microorganisms, which were finally confirmed again on the Api 20E gallery after 24 h of incubation at 37°C.

The French (AFNOR) FS V08-051 and Canadian (CCS: No.131) standards were used to define the microbiological criteria and to interpret the results obtained. The assessment criteria are as follows: 5.7 log_10_ CFU/g for total aerobic mesophils, 2.0 log_10_ CFU/g for total coliforms, 1.0 log_10_ CFU/g for *Enterobacteriaceae*, *Salmonella* absent in 25 g, *Staphylococcus aureus* absent in 1 g, and 2.0 log_10_ CFU/g for yeasts and molds.

### 2.4. Statistical Analysis

Analyses of variance and Tukey test multiple comparisons were performed with R software version 32 to calculate the means and standard deviations at the 5% significance level. The average microbial concentrations (average number of CFU/g) were calculated for each sample and compared to the limit values “*m*” and “*M*” set for foodstuffs [13]. “*m*” represents the number of CFU/g below which the sample is considered satisfactory. If the number of log_10_ CFU/g is between “*m*” and “*M*,” the sample is considered of acceptable quality, whereas samples containing more than “*M*” CFU/g are of unsatisfactory quality.

## 3. Results and Discussion

### 3.1. Comparison of the Physicochemical Quality of Liquid, Freeze-Dried, Hot Air-Dried, and Pasteurized Quail Eggs

#### 3.1.1. Comparison of the Water Activity of Liquid, Freeze-Dried, Hot Air-Dried, and Pasteurized Quail Eggs

**Figure 2:**
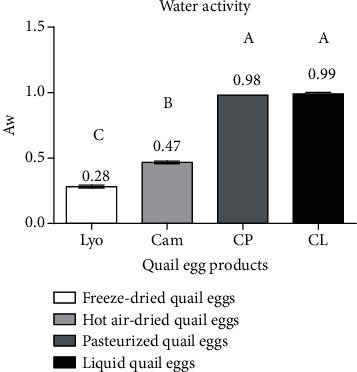
Water activity (Aw) of liquid (CL), freeze-dried (Lyo), hot air-dried (Cam), and pasteurized (CP) quail eggs.

Figure 2 shows the water activity values as a function of the types of quail egg products.

It is evident from Figure 2 that the water activity of freeze-dried quail eggs (0.28 ± 0.01) was the lowest, followed by that of hot air-dried quail eggs (0.47 ± 0.01). In contrast, the highest water activity was observed in the pasteurized (0.98 ± 0.00) and liquid (0.99 ± 0.01) quail eggs. A significant difference (*p* < 0.05) was observed between the water activity values of freeze-dried, hot air-dried, pasteurized, and liquid quail eggs except between those of pasteurized and liquid quail eggs. This difference is explained by the importance of the amount of available water evaporated from each egg product during processing. These values give each egg product its microbiological, oxidative, enzymatic, and nonenzymatic stability [14]. Thus, only freeze-dried and hot air-dried quail eggs are safe from microbial growth, lipid oxidation, and enzymatic and nonenzymatic reactions. This quality predicts that freeze-dried and hot air-dried quail eggs will have a long shelf life as opposed to pasteurized and liquid quail eggs with a short shelf life. In a study of the quality of solar-dried baobab fruits in Kenya, James et al. [15] found that the water activity of baobab pulp from formal processors ranged from 0.53 to 0.74 while that from informal processors ranged from 0.53 to 0.75.

#### 3.1.2. Comparison of the pH of Liquid, Freeze-Dried, Hot Air-Dried, and Pasteurized Quail Eggs

**Figure 3:**
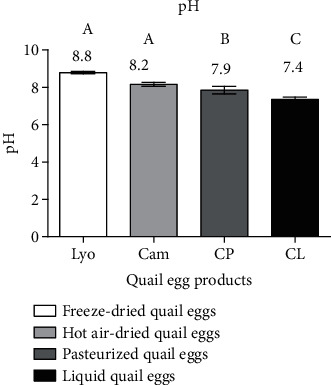
pH of liquid (CL), freeze-dried (Lyo), hot air-dried (Cam), and pasteurized (CP) quail eggs.

Figure 3 shows the pH values according to the types of quail egg products.

From Figure 3, freeze-dried quail eggs (8.8 ± 0.06) were more basic than hot air-dried quail eggs (8.2 ± 0.11), followed by pasteurized (7.9 ± 0.20) and liquid quail eggs (7.4 ± 0.12). The pH values of freeze-dried and hot air-dried quail eggs were significantly different (*p* < 0.05) from those of pasteurized and liquid quail eggs. The basicity of these egg products would be due to the amount of water evaporated during processing. These results are in agreement with those of Gonzalez Sanchez et al. [16] and Sousa et al. [17], who stated that the pH of egg products is between 7.5 and 9.6. On the other hand, the pH of these egg products complies with the European Economic Commission of the United Nations (EECUN) [9] standard, which stipulates a minimum pH of 7 for liquid eggs and 7.5 for dried eggs.

#### 3.1.3. Comparison of the Dry Matter Content of Liquid, Freeze-Dried, Hot Air-Dried, and Pasteurized Quail Eggs

**Figure 4:**
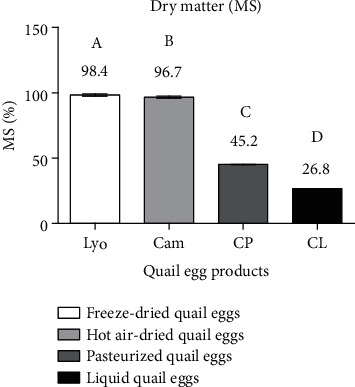
Dry matter content of liquid (CL), freeze-dried (Lyo), hot air-dried (Cam), and pasteurized (CP) quail eggs.

Figure 4 shows the dry matter content of quail egg products according to type.

Figure 4 reveals that freeze-dried (98.4 ± 0.78%) and hot air-dried (96.7 ± 0.76%) quail eggs had the highest dry matter contents compared to pasteurized (45.2 ± 0.06%) and liquid (26.8 ± 0.01%) quail eggs. A significant difference (*p* < 0.05) was observed between the dry matter values of all the evaluated quail egg products. This difference is due to the effectiveness of each processing technology in reducing the amount of available water in liquid quail eggs to the lowest possible level. The result of this work is similar to that of Kudre et al. [1] who found 98.92% dry matter in freeze-dried quail eggs. Similarly, the dry matter contents of liquid quail eggs are similar to those obtained by Dudusola [18] and Song et al. [19] who found 25.74%. Interestingly, the dry matter values of these egg products comply with the EECUN [9] standard which requires a minimum dry matter of 22% for liquid eggs and 95% for dried eggs. James et al. [15] showed that the dry matter contents of dried baobab pulp from formal processors ranged from 92.27% to 84.94% compared to 89.50% to 76.53% for informal processors.

#### 3.1.4. Comparison of the Fat Content of Liquid, Freeze-Dried, Hot Air-Dried, and Pasteurized Quail Eggs

**Figure 5:**
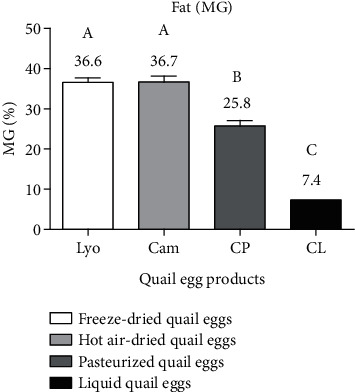
Fat content of liquid (CL), freeze-dried (Lyo), hot air-dried (Cam), and pasteurized (CP) quail eggs.

Figure 5 shows the fat content of the quail egg products produced.

From the data in Figure 5, freeze-dried (36.6 ± 1.13%) and hot air-dried (36.7 ± 1.46%) quail eggs were more concentrated in fat, while pasteurized (25.8 ± 1.33%) and liquid (7.4 ± 0.76%) quail eggs were less concentrated in fat. The fat values of freeze-dried, hot air-dried, pasteurized, and liquid quail eggs were significantly different (*p* < 0.05) from each other in contrast to freeze-dried and hot air-dried quail eggs which were statistically similar. These results are inconsistent with that of Kudre et al. [1] who quantified 2.16% lipid in freeze-dried quail eggs. This phase shift would be explained by the method used by Kudre et al. [1] which consisted of predegreasing liquid quail eggs by adding hexane before freeze-drying. The fat content of the liquid quail eggs was lower than that of Dudusola [18] and Song et al. [19] who obtained 11.91% in their respective studies. This difference in results is believed to be due to the feeding of the quail. The fat contents in the egg products were below the EECUN [9] standard, which requires a minimum of 9.8% for liquid eggs and 39% for dried eggs.

#### 3.1.5. Comparison of the Total Protein Content of Liquid, Freeze-Dried, Hot Air-Dried, and Pasteurized Quail Eggs

**Figure 6:**
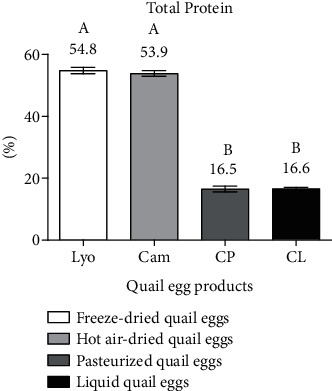
Total protein content of liquid (CL), freeze-dried (Lyo), hot air-dried (Cam), and pasteurized (CP) quail eggs.

Figure 6 shows the total protein content of liquid, freeze-dried, hot air-dried, and pasteurized quail eggs.

From Figure 6, it is evident that freeze-dried (54.8 ± 0.98%) and hot air-dried (53.9 ± 0.93%) quail eggs held the highest protein values against those of liquid (16.6 ± 0.47%) and pasteurized (16.5 ± 0.95%) quail eggs which were low. Thus, the protein content of freeze-dried quail eggs and those of hot air-dried eggs were superior (*p* < 0.05) to those of liquid quail eggs and pasteurized quail eggs. This trend is due to the concentration of protein in freeze-dried and air-dried quail eggs caused by water removal. The protein contents in the egg products were above those of the EECUN standard [9], which requires a minimum of 10.5% for liquid eggs and 45% for dried eggs. The results obtained are lower than those of Kudre et al. [1] who found a value of 94.17% for the protein content of freeze-dried quail eggs. The total protein content of 11.98% according to the respective works of Dudusola [18] and Song et al. [19] is lower than the results of liquid quail eggs. This difference is considered to be related to the methods of freeze-drying and protein determination and feeding of quails. Baniel et al. [20] and Segura-Campos et al. [21] reported that spray-dried hen egg white and air-dried quail egg white contained 90% protein powder and 93.96% protein powder, respectively.

#### 3.1.6. Comparison of the Crude Ash Content of Liquid, Freeze-Dried, Hot Air-Dried, and Pasteurized Quail Eggs

**Figure 7:**
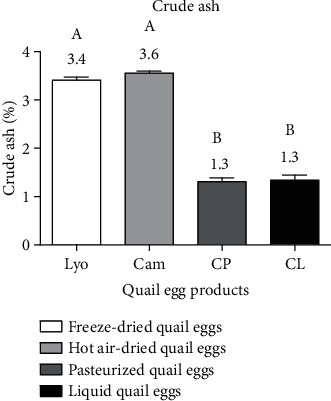
Crude ash content of liquid (CL), freeze-dried (Lyo), hot air-dried (Cam), and pasteurized (CP) quail eggs.

Figure 7 shows the crude ash contents of liquid, freeze-dried, hot air-dried, and pasteurized quail eggs.

Regarding Figure 7, the highest crude ash values were observed in freeze-dried (3.4 ± 0.06%) and hot air-dried (3.6 ± 0.04%) quail eggs, while the lowest crude ash values were obtained in liquid (1.3 ± 0.10%) and pasteurized (1.3 ± 0.08%) quail eggs. The crude ash content of freeze-dried and hot air-dried quail eggs differed (*p* < 0.05) from liquid quail eggs and pasteurized quail eggs. These different values obtained would be due to the concentration of dry matter contained in each egg product. These results are in agreement with those obtained by Krudre et al. [1] which were 3.23% for freeze-dried quail eggs. The ash content of 1.04% for liquid quail eggs determined by Dudusola [18] and Song et al. [19] is similar to the results obtained for liquid quail eggs.

### 3.2. Comparison of Microbiological Quality of Liquid, Pasteurized, Hot Air-Dried, and Freeze-Dried Quail Eggs

**Figure 8:**
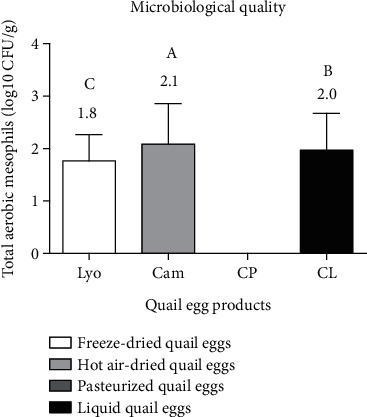
Microbiological quality of liquid (CL), freeze-dried (Lyo), hot air-dried (Cam), and pasteurized (CP) quail eggs.

Figure 8 shows the microbiological quality of liquid, freeze-dried, hot air-dried, and pasteurized quail eggs.

From Figure 8, only Total Aerobic Mesophils (TAM) were detected in the liquid, hot air-dried, and freeze-dried quail eggs, but not in the pasteurized quail eggs. The microbial loads in TAM ranged from 1.8 ± 1.0 log_10_ CFU/g to 2.1 ± 1.5 log_10_ CFU/g, with 2.0 ± 1.4 log_10_ CFU/g for liquid quail eggs, 1.8 ± 1.0 log_10_ CFU/g for freeze-dried quail eggs, and 2.1 ± 1.5 log_10_ CFU/g for hot air-dried. These different values were much lower than the minimum value set by the French standard (FS V08-051) which is 5.7 log_10_ CFU/g for all egg products. The liquid, pasteurized, hot air-dried, and freeze-dried quail eggs were of highly satisfactory hygienic quality (100%) for total aerobic mesophils. In other words, liquid, pasteurized, freeze-dried, and air-dried quail eggs were all fit for consumption. Total coliforms (0 CFU/g), *Enterobacteriaceae* (0 CFU/g), yeasts (0 CFU/g), molds (0 CFU/g), *Salmonella*, and *Staphylococcus aureus* were all absent in the quail egg products analyzed, while French (FS V08-051) and Canadian (CCS: No.131) standards provided 2.0 log_10_ CFU/g for total coliforms, 1.0 log_10_ CFU/g for *Enterobacteriaceae*, *Salmonella* absent in 25 g, *Staphylococcus aureus* absent in 1 g, and 2.0 log_10_ CFU/g for yeasts and molds. The liquid, pasteurized, hot air-dried, and freeze-dried quail eggs were all in compliance with French (FS V08-051) and Canadian (CCS: No.131) standards with a highly satisfactory hygienic quality of 100%, therefore all fit for consumption. The activities of microorganisms in an egg product depend on the nature of the egg product, its initial level of contamination, and especially the temperature [22, 23]. James et al. [15] found that baobab pulp from formal processors had significantly lower loads of *Enterobacteriaceae*, yeasts, and molds than pulp from informal processors. In respect of the various standards, quail egg products can then be used for any purpose without the risk of food contamination. Nevertheless, there is a significant difference (*p* < 0.05) between the TAM loadings of egg products from the different transformation processes studied. Then, the TAM load of freeze-dried quail eggs was lower than that of liquid quail eggs. Therefore, freeze-drying reduced the TAM load of liquid quail eggs. In contrast to freeze-dried quail eggs, hot air-dried quail eggs had a higher TAM load than liquid quail eggs. The increase of the TAM load of hot air-dried quail eggs compared to liquids would be due to their contamination during the powdering process favored also by the decrease of their temperature. The total absence of microbial load in the pasteurized quail eggs is explained by the effectiveness of the hot conditioning and coupled with the effectiveness of time/temperature. Good control of the majority of pathogenic species and in particular of *Salmonella* in egg products is ensured by respecting the rules of hygiene and pasteurization [22]. From a microbiological point of view, pasteurization was more effective than freeze-drying and hot air-drying.

## 4. Conclusion

From this study, it is important to note that freeze-dried quail eggs had the best physicochemical quality, which differed from that of hot air-dried quail eggs due to water activity and dry matter content. Freeze-dried and hot air-dried quail eggs can guarantee better preservation of quail eggs for a long time. The pasteurized eggs can be preserved for a short duration but can stay longer than the liquid eggs. Microbiologically, pasteurization was more effective followed by freeze-drying and hot air-drying, but all quail egg products were overall of highly satisfactory hygienic quality. Hot air-drying and pasteurization can substitute freeze-drying for the processing of quail eggs despite the difference in the physicochemical quality of the pasteurized quail eggs.

## Data Availability

The original research generated data used to support the findings of this study are included within the article.
